# Supplementary material for: The Benefits of Radical Treatments with Synchronous Splenectomy for Patients with Hepatocellular Carcinoma and Portal Hypertension
Source: Cancers (Basel). 2022 Jun 28;14(13):3155. doi: 10.3390/cancers14133155 (PMC9264870; doi:10.3390/cancers14133155)
Supplement: Supplementary file 1 [file cancers-14-03155-s001.zip › cancers-1770442-supplementary.pdf]

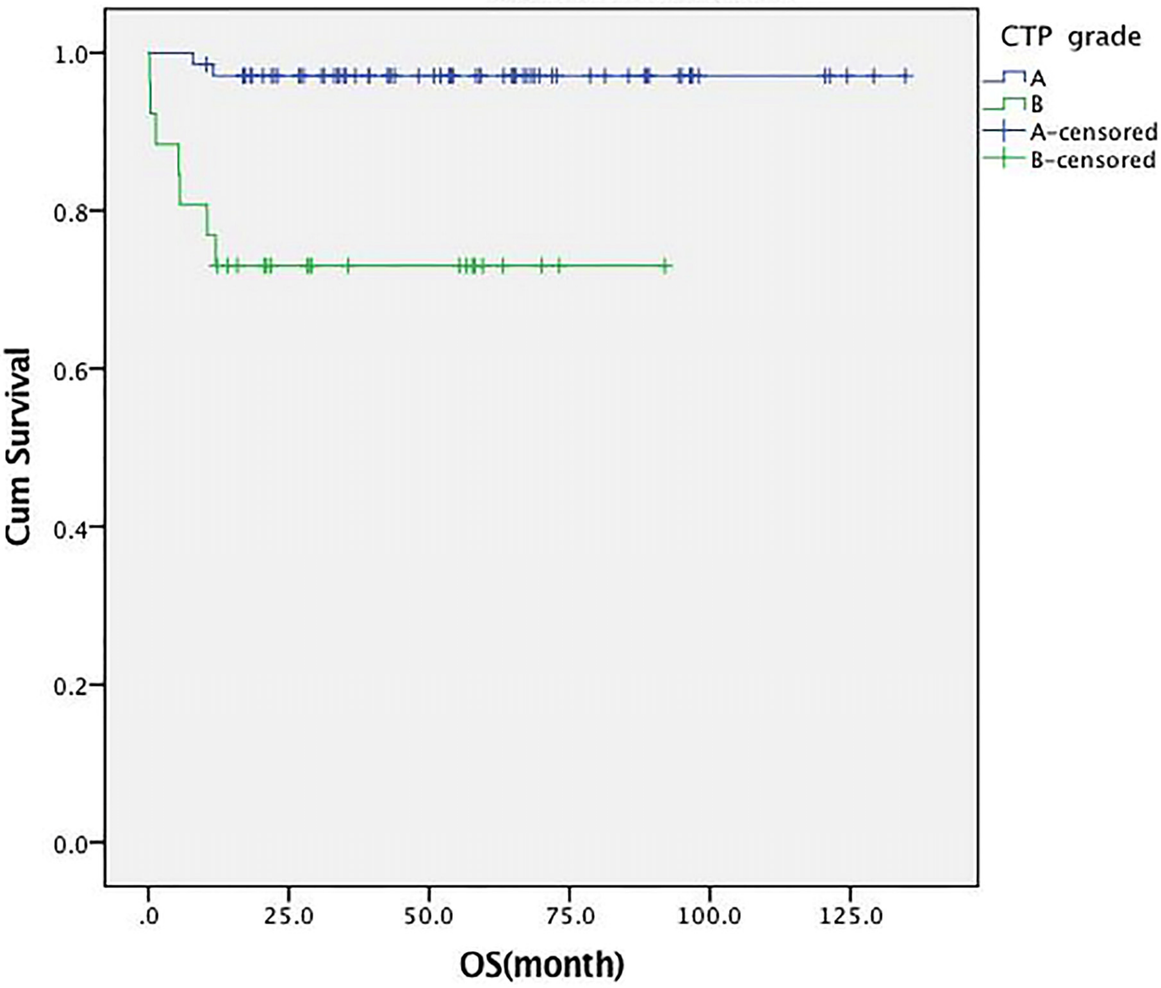

Figure S1: Different CTP grade at 1-year OS in the HS group ($p = 0.000$).

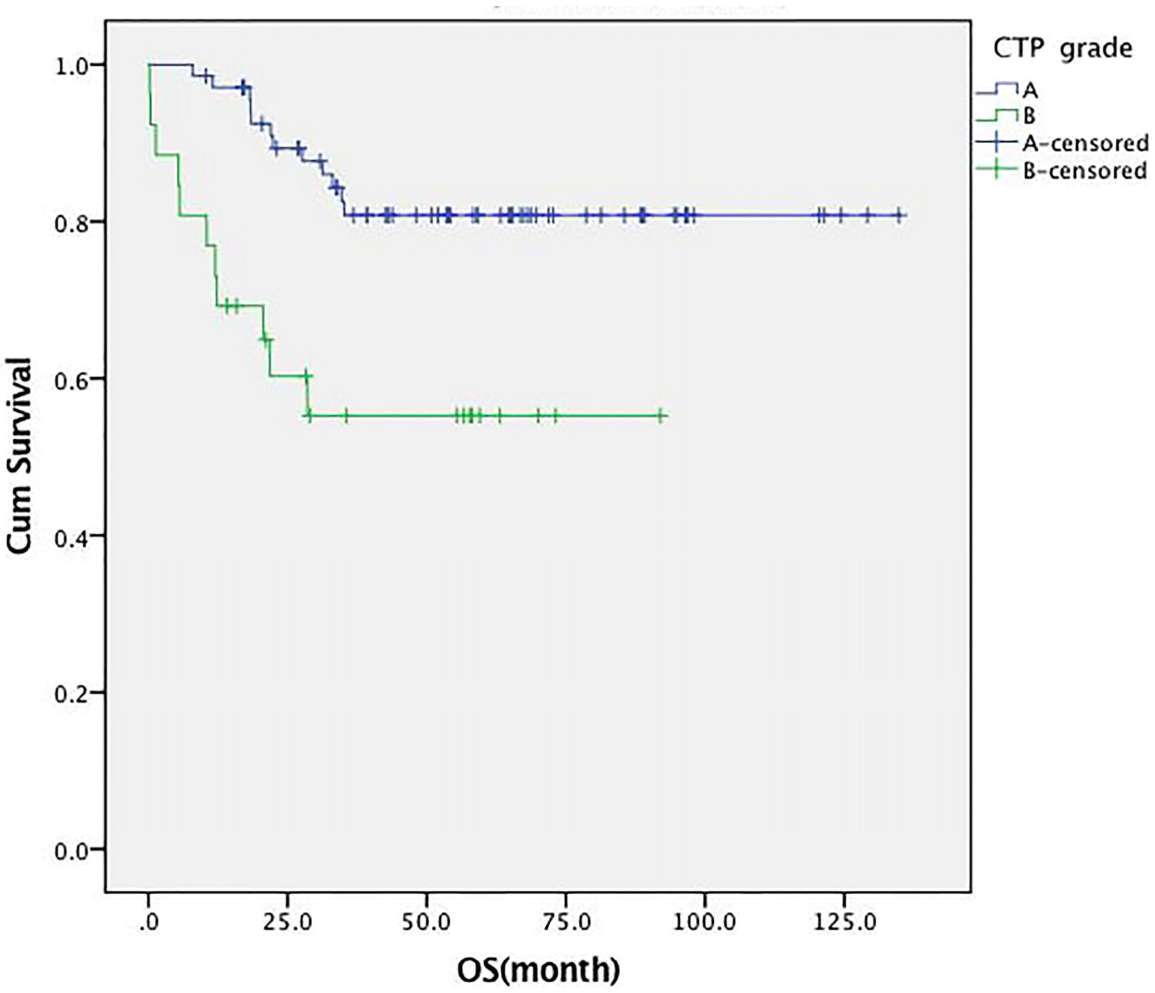

Figure S2: Different CTP grade at 3-year OS in the HS group ($p = 0.002$).

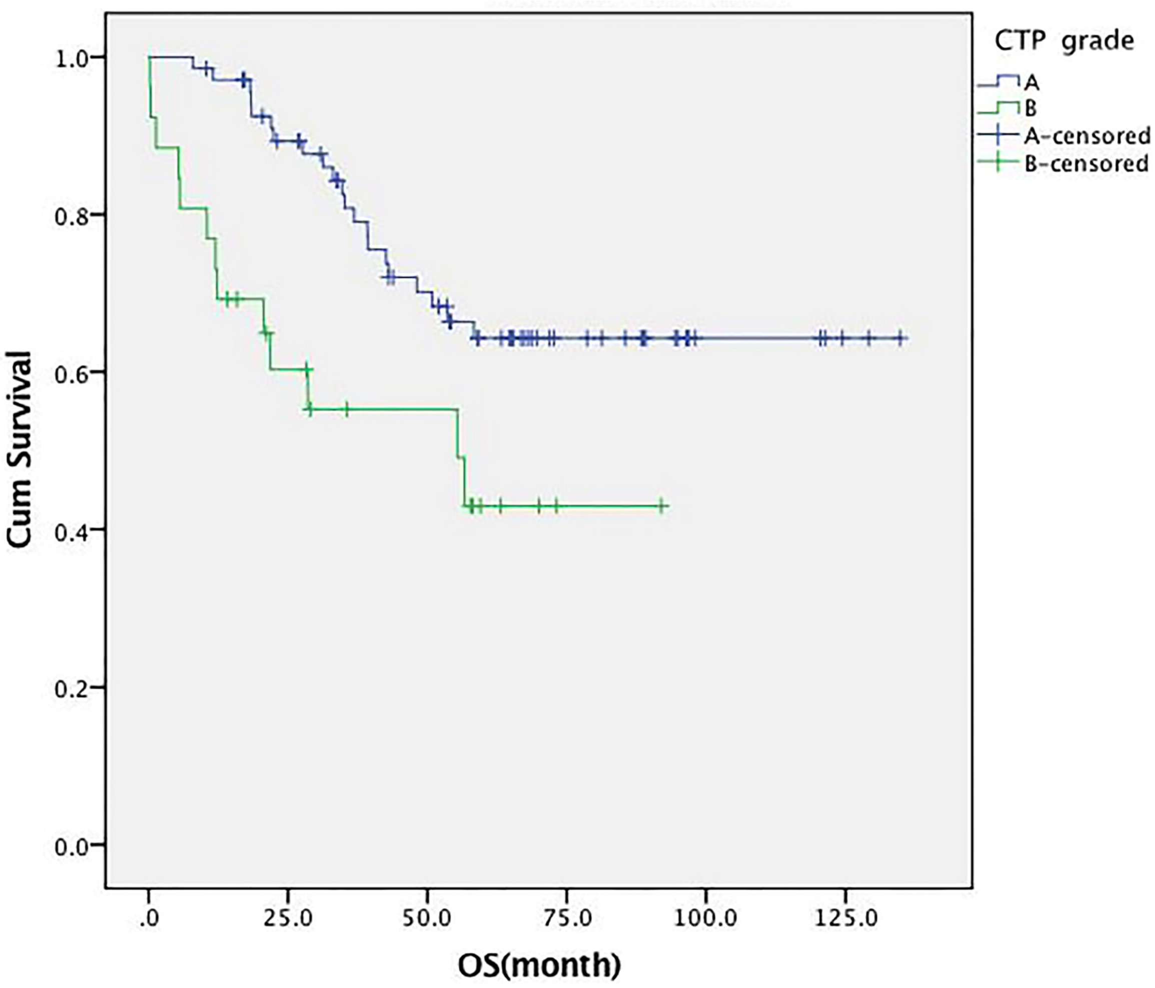

Figure S3: Different CTP grade at 5-year OS in the HS group (p = 0.009).

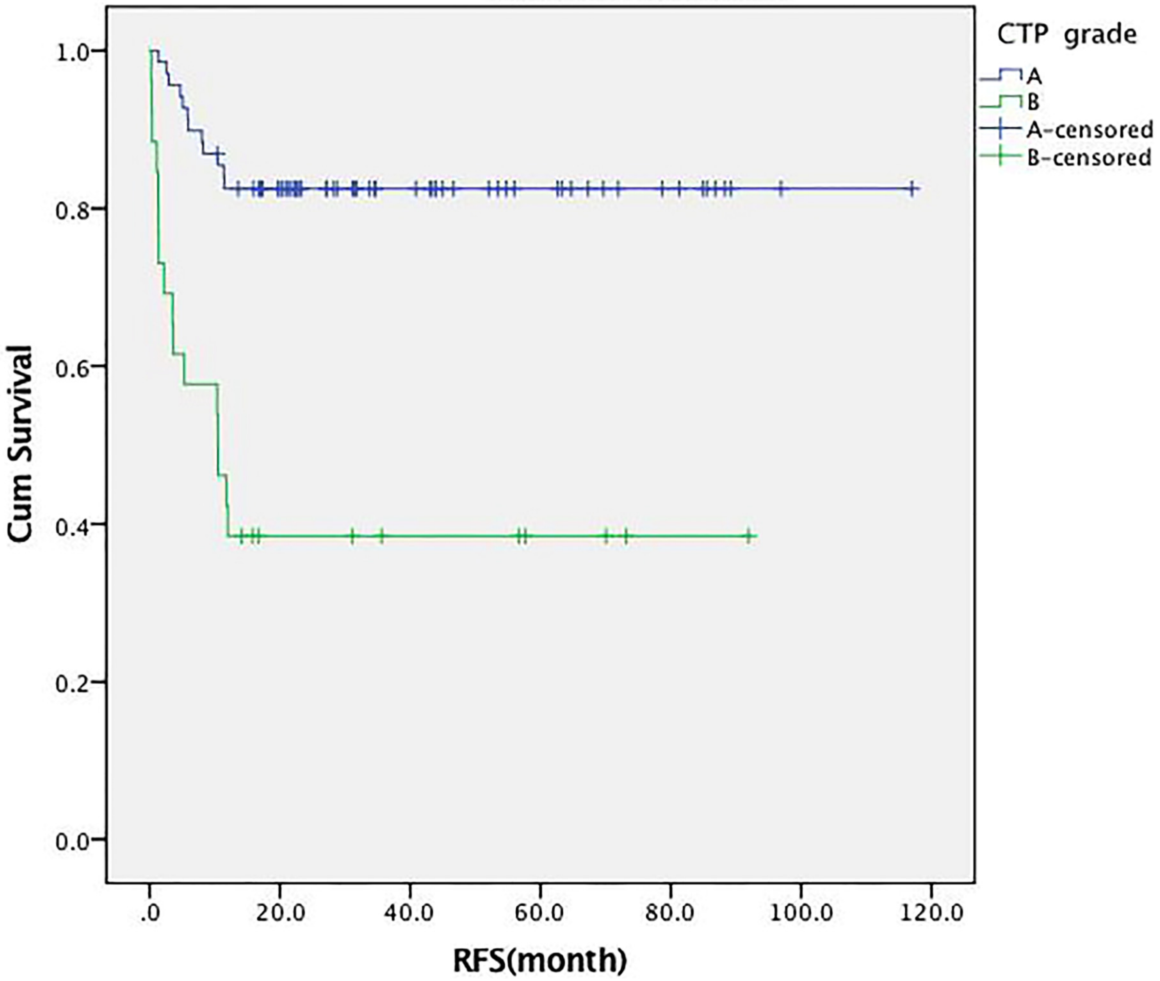

Figure S4: Different CTP grade at 1-year RFS in the HS group (p = 0.000).

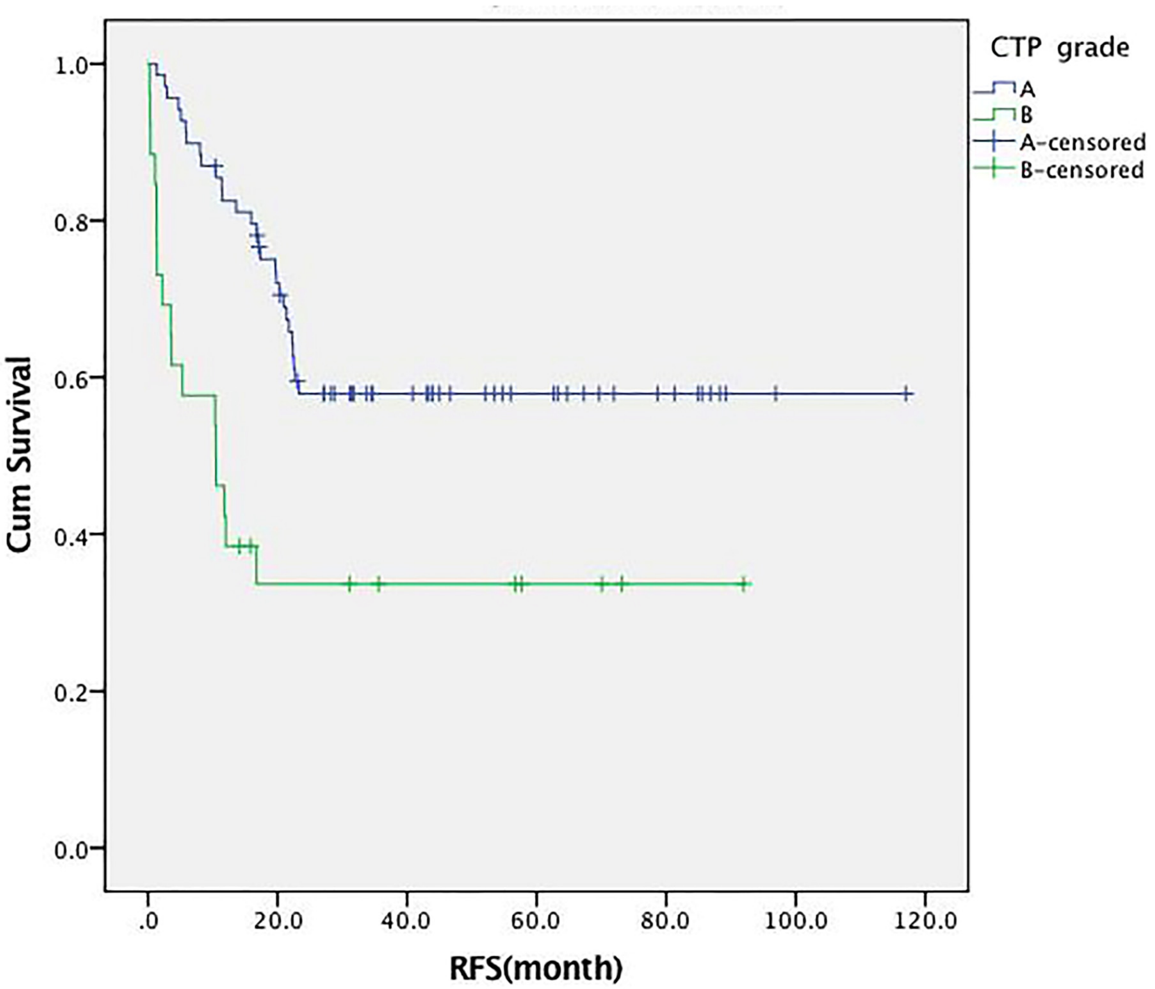

Figure S5: Different CTP grade at 2-year RFS in the HS group (p = 0.001).

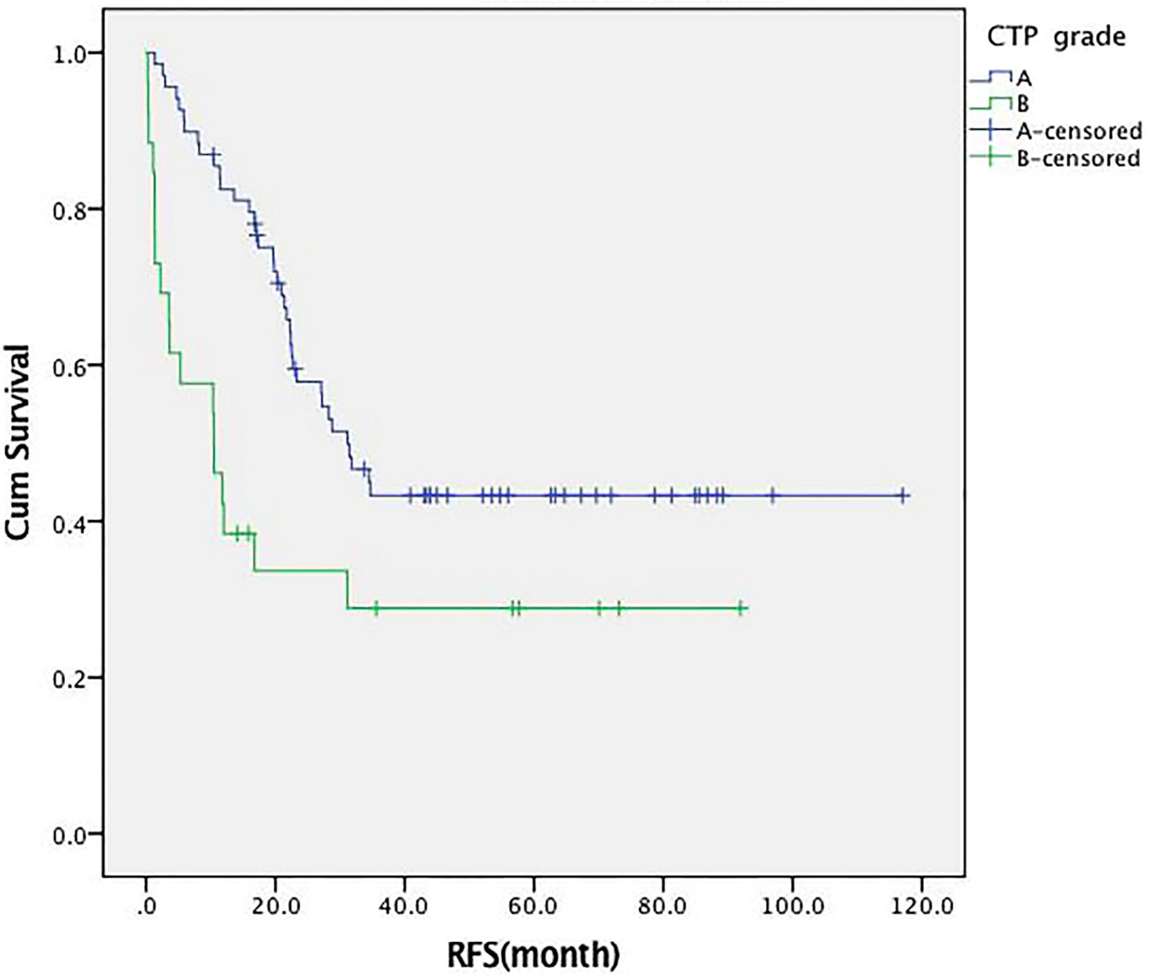

Figure S6: Different CTP grade at 3-year RFS in the HS group (p = 0.004).

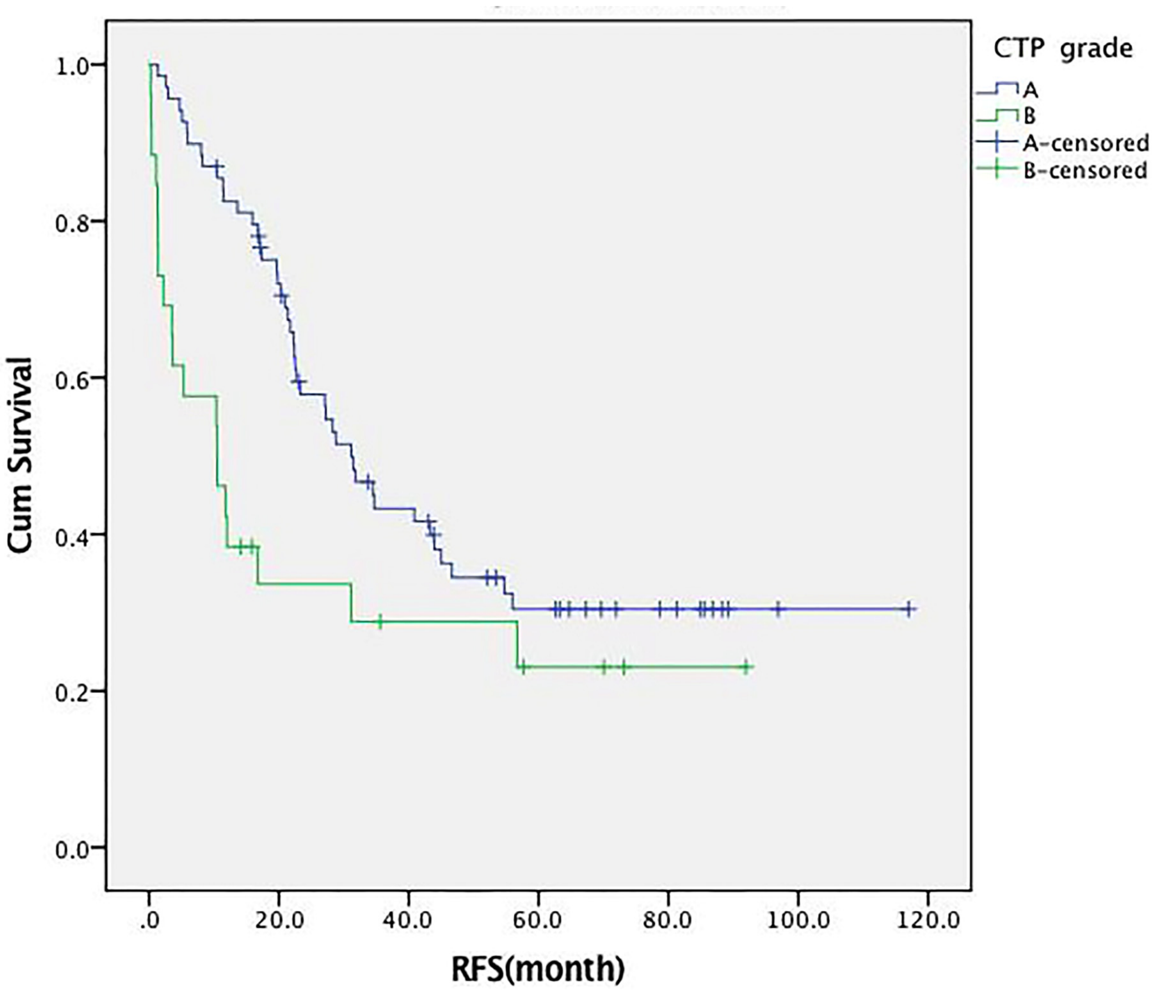

Figure S7: Different CTP grade at 5-year RFS in the HS group (p = 0.014).
